# Pragmatic Neurorehabilitation Approach for Improving Quality of Life in Duchenne Muscular Dystrophy: A Case Report

**DOI:** 10.7759/cureus.56315

**Published:** 2024-03-17

**Authors:** Radha Nangliya, Anam R Sasun, Snehal Samal

**Affiliations:** 1 Department of Neurophysiotherapy, Ravi Nair Physiotherapy College, Datta Meghe Institute of Higher Education and Research, Wardha, IND

**Keywords:** dystrophin, pediatrics rehabilitation, knee-ankle-foot orthosis, gower’s sign, duchene muscular dystrophy

## Abstract

This case report provides insights into the physiotherapy management of a 12-year-old male with Duchenne muscular dystrophy (DMD). DMD is a devastating genetic disorder characterized by progressive muscle degeneration and weakness. Skeletal muscle degeneration is induced by a genetic disorder. It is a common X-linked condition that causes hypertrophy of the calves and proximal muscular weakness in children. It frequently results in early mortality, wheelchair confinement, and delays in motor development. Physiotherapy interventions aim to optimize functional abilities and quality of life in individuals with DMD. This case report highlights the effectiveness of physiotherapy in managing DMD progression. This study presents a case exhibiting notable clinical symptoms, highlighting the urgency for advanced treatments to combat this debilitating disease. Outcome measures such as body mass index, spirometry, manual muscle testing, and the World Health Organization Quality-of-Life scale are used to report patient progress. The treatment plan was carried out for six weeks, five times a week. Physiotherapy strategies include diet management, stretching and splinting techniques, and pulmonary training. While current treatments focus on symptom management, ongoing research holds promise for the development of more effective therapies to improve outcomes and quality of life for affected individuals. Multidisciplinary care, including neurophysiotherapy rehabilitation, plays a crucial role in managing the symptoms and complications of DMD, emphasizing the importance of comprehensive support for patients and their families. At the end of our rehabilitation, the patient showed significant improvement in the outcome measures.

## Introduction

Duchenne muscular dystrophy (DMD) is a progressive muscle-withering disease. The patients first exhibit symptoms at two to three years of age, which include trouble climbing stairs, a waddling gate, calf pseudohypertrophy, proximal muscle weakness, and frequent falls [1]. Most people start to need assistance breathing around the age of 20, and by the time they reach 10 or 12 years old, they become dependent on wheelchairs. The most common neuromuscular disease affects up to 1,600 male infants born globally. The X chromosome's dystrophin gene mutations are the source of it, and the clinical symptoms do not appear at birth. Generally, the first symptoms show at four years old, which is the typical age of diagnosis [2]. As the condition worsens, people with DMD need more help moving around and usually stop being able to walk on their own by the time they are in their early teens, which makes them dependent on wheelchairs [3].

Mutations in the dystrophin gene are the cause of it. This causes injury to the muscles and inadequate repair of proteins, which increases the wasting and degeneration of the muscles. The cycle of damage and repair, which frequently begins by the time a child is three or four years old, eventually leads to the replacement of muscle by fibrofatty tissue [4,5]. Three distinct promoters in the brain, muscle, and Purkinje cerebellar neuron create three full-length isoforms of the DMD gene; however, alternative splicing events lead to the creation of countless other isoforms [6]. Boys that have Gower's sign have trouble running and toe walking. They have lumbar lordosis, calf hypertrophy, and a waddling gait. Because their lower limb's proximal muscles are weak, they must walk up using their hands and arms [7]. Elevated serum creatine kinase (CK) levels, muscle biopsy, electromyography, and genetic analysis are all used in the diagnosis of DMD. Elevated blood CK levels are the consequence of protein leakage caused by damaged sarcolemma. There is also an increase in other enzymes such as lactate dehydrogenase, aldolase, aspartate transaminase, and alanine transaminase [8].

Physiotherapy rehabilitation aims to prevent complications such as contracture development, preserve motor functions of muscles, maintain lung volume, provide effective teaching, and assist with coughing [9]. Physiotherapy is important to encourage walking and prevent joint abnormalities. Knee-ankle-foot orthoses (KAFOs), which effectively extend walking for an average of 18 months to two years, are offered as a form of rehabilitation to patients with DMD. The process, which makes use of KAFO that is custom constructed, is usually well tolerated. Previously, to reduce ankle rigidity and facilitate KAFO fitting, surgical relaxation of the Achilles tendon was necessary. Recent research has demonstrated that, in many situations, repeated casting of the ankles can be administered in place of surgically releasing the Achilles tendons [10]. Early clinical studies show that resistance exercise without isometric focus does not cause physical deterioration in DMD. Strength training has shown improvements without overloading weakness. However, there's insufficient evidence to support or refute the use of strengthening exercises in boys with DMD. Further research is needed to ensure the safety of strength training in DMD [11]. Submaximal exercise may have limited value in increasing strength in DMD [12]. Whole-body vibration training is a potential supportive therapy for neuromuscular illnesses since it is utilized to increase muscle strength and function [13].

## Case presentation

Patient information

A 12-year-old male reported to a neurophysiotherapy rehabilitation center with complaints of difficulty breathing for two months and generalized weakness for over five years. Other associated complaints are a repeated history of falls, fatigue, and an inability to walk or climb stairs. Medical history revealed the presence of similar symptoms in the elderly elder brother, who is now bedbound. History revealed that the patient started toe-walking at the age of five years. The child had an obese appearance.

Clinical examination

**Figure 1:**
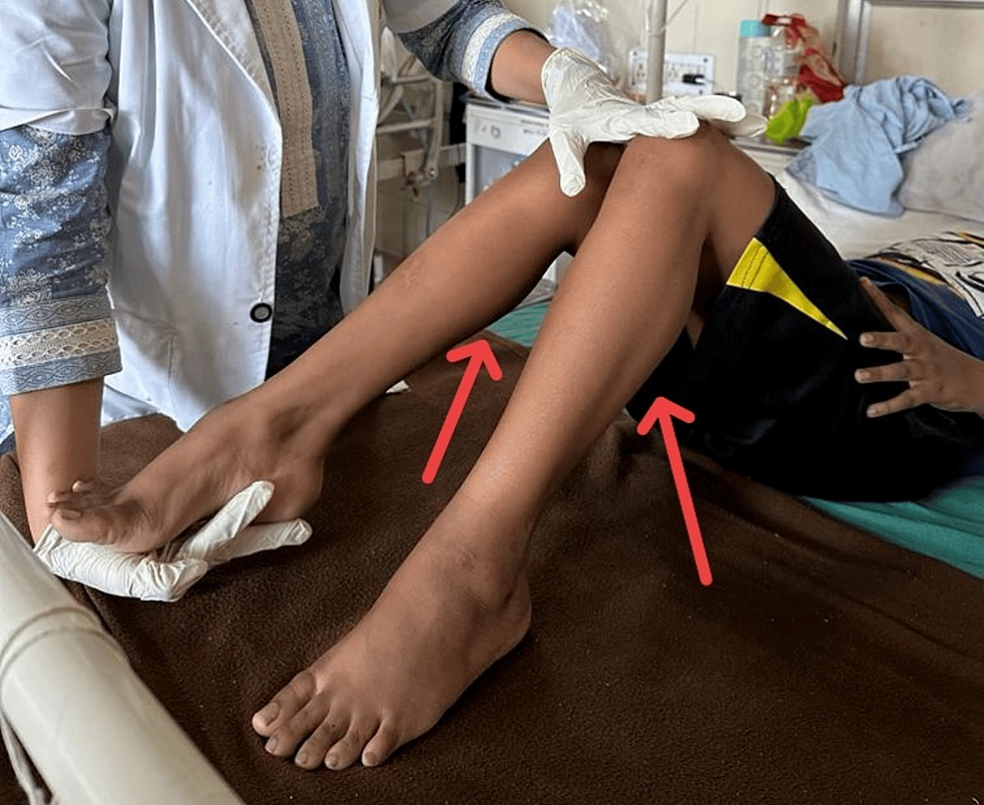
Calf muscle hypertrophy.

A physiotherapy assessment was done after obtaining proper oral consent from the patient and relatives. On observation, the child has an endomorphic build, calf hypertrophy on bilateral legs, and bilateral knee flexion contracture. The bilateral ankle demonstrated Pes Planus. The patient showed a positive grower’s sign. Along with toe-walking and lumbar hyperlordosis, the patient also displayed a waddling gait. Bilateral lower and upper limb muscle strength is reduced. During the intraoral examination, findings included an anterior open bite, left posterior crossbite, swollen tongue, crowding in the lower anterior region, decay in tooth 46, and poor dental hygiene status. Examination of the reflexes revealed reduced deep and superficial responses. Girth measurement revealed calf hypertrophy (Figure 1). The biochemistry report is given in Table 1. Muscle tone exhibited hypotonia upon examination and palpation (Table 2). The girth measurement is shown in Table 3.

**Table 1:** Biochemistry report. U/L, units per liter

Biochemistry	Values	Normal ranges
CreatinekKinase	20.232 U/L	55-170 U/L
Alanine aminotransferase	208 U/L	<33 U/L
Aspartate aminotransferase	190.5 U/L	<32 U/L
Lactate dehydrogenase	6470 U/L	<250 U/L

**Table 2:** Muscle tone. NA, not assessable; TGS, tone grading scale

Tone (According to TGS)	Right side	Left side
Upper limb		
Shoulder flexors	1+	1+
Shoulder abductors	1+	1+
Elbow flexors	1+	1+
Wrist flexors	1+	1+
Finger flexors	1+	1+
Lowe limb		
Hip flexors	1+	1+
Hip abductors	1+	1+
Knee flexors	NA	NA
Ankle dorsiflexors	1+	1+
Ankle plantarflexors	1+	1+

**Table 3:** Girth measurement of the upper limb and the lower limb.

Area	Right	Left
Upper arm	20 cm	20 cm
Thigh	29 cm	29 cm
Calf	23 cm	23 cm

Diagnostic assessment

Biochemistry analysis showed elevated levels of CK (20.232 U/L), alanine aminotransferase (208 U/L), aspartate aminotransferase (190.5 U/L), and lactate dehydrogenase (647 U/L). The muscle biopsy report showed extensive loss of skeletal muscle fibers that were replaced by fat tissue, and extensive fibrosis was noted. Electromyography revealed a myopathic pattern in the right vastus lateralis and deltoid muscle, suggestive of primary muscle disease. Based on history, clinical examination, and investigations, the diagnosis of DMD was established.

Therapeutic interventions

Once the diagnosis of the disease has been done, the physiotherapists need to be involved in multi-disciplinary rehabilitation, to guide the parents through multiple stages of the disease. The treatment of muscular dystrophy is impossible without the parents' cooperation. The physiotherapists need to assess the agreed treatment at regular intervals to ensure everything is progressively correct and be able to plan for the next stage.

Diet Management

It is critical to keep an eye on the patient's weight. Gaining weight is frequent as mobility decreases, and it exacerbates the loss of ambulation. Consumption of salt and calories was monitored. Adequate intake of calcium and vitamin D was ensured. A high-fiber, well-hydrated diet was initiated.

Strength Training

Resisted proprioceptive neuromuscular facilitation (PNF) exercises for affected muscles are beneficial. Exercises using balance and strengthening techniques in standing, sitting, and lying were given. Proprioceptive neuromuscular patterns, specifically D1-D2 flexion extension for bilateral upper and lower limbs were done. Rhythmic initiation pattern was commenced. The dosage involved 10 repetitions, two sets with proper rest intervals. The progression of the regimen began with rhythmic initiation followed by concentric and eccentric exercises.

Stretching and Splinting

Stretching exercises were performed for shoulder flexors, abductors, elbow flexors and extensors, wrist flexors, hip flexors, abductors, knee flexors, and the Achilles tendon, with a hold time of 20 seconds and five repetitions. Stretching was implemented to prevent the development of contractures. The stretching was done within a full range of motion. Passive stretching and splinting were given for the ankle. Ankle-foot orthoses were provided to maintain the ankle in a neutral position.

Pulmonary Training

Respiratory training is essential to preserve lung function over time. Respiratory muscle weakness leads to weak cough expectoration and airway clearance impairment. Mucus mobilization devices like flutter devices and oscillating positive expiratory pressure were used. Other chest physiotherapy techniques such as manual percussion, vibration, and postural drainage techniques were used. Each technique was given for 20 reps, two sets. Exercises for breathing such as diaphragmatic breathing and pursed lip breathing were taught.

Minimal Weight-Bearing

Standing is essential for controlling DMD since it lessens the severity of contractures, the development of decubitus ulcers, and the curvature of the spine. It enhances the functions of the gastrointestinal tract, respiratory system, and bone mineral density. Twice a day, standing frames were utilized efficiently.

Access to Wheelchair Services

Energy conservation techniques were commenced to avoid undue fatigue to muscles. For people with DMD, the availability of effective and timely adjustments and assistive technology improves their quality of life. The family's overall quality of life is severely harmed by delays in the provision of essential services. For an independent and productive life, suitable housing adaptations, electric beds and wheelchairs, internet connectivity, and computer use are necessary, but adults with DMD can lead wonderful lives with the right support.

Follow-up and outcome measures

Table 4 shows the patient's World Health Organization Quality of Life (WHO-QOL) scale scores for a 12-year-old child with DMD before and after rehabilitation. Table 4 presents outcome measures, including the range of motion (in degrees) for various joints, active range of motion, and muscle strength (rated out of 5). The progression of the patient's condition is depicted in Tables 5-6.

**Table 4:** Outcome measures of the rehabilitation. WHO-QOL, World Health Organization Quality-of-Life scale; cc, cubic centimeters

Outcome measures	Pre-intervention	Post-intervention
WHO-QOL	40/100	70/100
Body mass index	Obesity class 1	Overweight
Spirometer	900 cc	1,200 cc

**Table 5:** Active range of motion of joints.

Active range of motion	Pre-rehabilitation		Post-rehabilitation	
	Right	Left	Right	Left
Joint movement				
Shoulder flexion	100	100	160	160
Shoulder abduction	100	100	160	160
Shoulder rotation	30	30	35	35
Elbow flexion	150	50	170	170
Wrist flexion	20	20	30	30
Lower limb				
Hip flexion	0	0	20	20
Hip extension	0	0	20	20
Hip abduction	0	0	0	0
Knee flexion	50 (flexion deformity)	50 (flexion deformity)	70	70
Ankle dorsiflexion	10	10	15	15
Ankle plantarflexion	20	20	30	30

**Table 6:** Manual muscle testing.

	Pre-rehabilitation		Post-rehabilitation	
	Right	Left	Right	Left
Shoulder flexors	3/5	3/5	3/5	3/5
Shoulder abductors	3/5	3/5	3/5	3/5
Elbow flexors	3/5	2/5	3/5	3/5
Wrist flexors	3/5	2/5	3/5	3/5
Hip flexors	1/5	1/5	2/5	2/5
Hip abductors	1/5	1/5	2/5	2/5
Knee flexors	1/5	1/5	2/5	2/5
Ankle plantarflexors	1/5	1/5	2/5	2/5
Ankle dorsiflexors	1/5	1/5	2/5	2/5

## Discussion

Mutations in the dystrophin gene, which stop the body from producing the protein dystrophin needed for muscle contraction, result in DMD, a prevalent form of muscular dystrophy. This leads to the deterioration and progressive loss of muscle mass as fibrofatty tissue eventually replaces the damaged muscle tissue. Mutations in the DMD gene can result in an unstable and prematurely shortened dystrophin protein. Sixty-seven percent of patients with DMD have intragenic deletions as their predominant mutation. Degeneration of muscle fibers, instability of the membrane, and heightened vulnerability to mechanical stress are the results of dystrophin deficiency. The absence of dystrophin from the plasma membrane disrupts the cytoskeleton, causes delocalization of dystrophin-associated proteins, and increases mechanical stress sensitivity. High serum marker levels of creatinine kinase, muscle biopsy, electromyography, and genetic studies are used to diagnose DMD.

Essentially, effective weight management principles build upon strategies used for preventing obesity. Family involvement is crucial, with interventions encompassing both nutritional and psychosocial support, along with regular follow-ups. They may include structured meal plans with self-monitoring, supervised by a dietitian trained in weight management, aiming to avoid overly restrictive diets. A multidisciplinary team comprising a registered dietitian, a physical activity specialist, and a mental health professional is essential for comprehensive support [14]. Gene therapy and stem cell therapy are two modern forms of treatment that imply dystrophin-like protein overexpression. Genetic testing and prenatal counseling are being used to give patients with DMD hope for a longer and higher quality of life [15]. DMD is a chronic disease requiring multidisciplinary and integrative care, often requiring a pediatric neurologist to coordinate care. Due to a lack of facilities, muscle biopsy for the dystrophin assay was difficult, but recent developments in mutational analysis have improved its accuracy. Children with DMD often experience depression, social isolation, and poor school performance [16]. Parents' emotional burnout due to DMD's worsening muscle weakness and poverty may have led to a refusal to follow up [17]. According to Nishizawa et al., standing motor function improved significantly in some patients in the sufficiently wearing group, suggesting that wearing night splints may promote and/or maintain standing motor function in patients with DMD [18].

The study analyzed the identification and diagnosis of a 400-person DMD population, primarily boys, with onset in early childhood and walking difficulties. The study found no significant effect of diet on clinical symptoms. Most patients attributed DMD to consanguine marriages, but genetic confirmation or a primary diagnostic pattern was lacking. Secondary dystrophy confirmation was done through electromyography and muscle biopsies, but only 55% of the population underwent invasive testing, hindering clinical trials and research advancements [19]. According to a study, boys with DMD who engage in moderate-intensity isometric exercise do not experience acute muscle injury. Additionally, the study discovered that a program of moderate-intensity isometric strengthening exercises is safe and helps ambulatory boys increase their strength and function. The study offers quantifiable proof of elevated peak torque and enhanced function, in addition to corroborated reports of stronger muscles following resistive workout regimens. According to the study, patients with DMD may benefit from and feel safe during correctly dosed strengthening exercises [20]. The significance of the study's results is in expanding our knowledge of the function of strengthening exercises in the treatment of DMD. It highlights the practicability, effectiveness, and safety of these kinds of exercises, laying the groundwork for future studies and the creation of focused rehabilitation plans for people with DMD [21].

## Conclusions

This case study offers insightful information about the function of neurophysiotherapy rehabilitation in managing the many problems related to DMD. It highlights the significance of a comprehensive and individualized approach to care, with the ultimate goal of enhancing the functional independence and general well-being of those afflicted with this crippling genetic illness. Our rehabilitation strategies worked with the parent's cooperation. Our rehabilitation consisted of working to manage the weight and obesity of the patient by regulating his calorie, salt, water, and fiber intake. Pulmonary rehabilitation worked to improve the lung volume of the patient by using modern devices like flutter, spirometer, and oscillating positive expiratory pressure. Splinting and other interventions significantly contributed to improving functional capacity. The physiotherapists need to assess the agreed-upon treatment at regular intervals to ensure everything is progressively correct and be able to plan for the next stage.
